# New Books

**Published:** 2006-08

A Basic Introduction to Pollutant Fate and Transport: An Integrated Approach with Chemistry, Modeling, Risk Assessment, and Environmental Legislation

Frank M. Dunnivant, Elliot Anders

Hoboken, NJ:John Wiley & Sons, 2006. 504 pp. ISBN: 0-471-65128-1, $89.95

Atmospheric Chemistry and Physics: From Air Pollution to Climate Change, 2nd Ed.

John H. Seinfeld, Spyros N. Pandis

Hoboken, NJ:John Wiley & Sons, 2006. 1232 pp. ISBN: 0-471-72017-8, $150

Cancer Diagnostics with DNA Microarrays

Steen Knudsen

Hoboken, NJ:John Wiley & Sons, 2006. 208 pp. ISBN: 0-471-78407-9, $125

Chemical Genomics: Small Molecule Probes to Study Cellular Function

Stefan Jaroch, Weinmann Hilmar, eds.

New York:Springer, 2006. 174 pp. ISBN: 3-540-27865-6, $79.95

Comparative Environmental Politics

Jerry McBeath, Jonathan Rosenberg

New York:Springer, 2006. 193 pp. ISBN: 1-4020-4762-2, $125

Ecological Risks Associated with the Destruction of Chemical Weapons

Vladimir M. Kolodkin, Wolfgang Ruck, eds.

New York:Springer, 2006. 340 pp. ISBN: 1-4020-3136-X, $65

Environmental UV Radiation: Impact on Ecosystems and Human Health and Predictive Models

Francesco Ghetti, Giovanni Checcucci, Janet F. Bornman, eds.

New York:Springer, 2006. 288 pp. ISBN: 1-4020-3696-5, $79.95

Genomics and Proteomics Engineering in Medicine and Biology

Metin Akay, ed.

Hoboken, NJ:John Wiley & Sons, 2006. 336 pp. ISBN: 0-471-63181-7, $98.95

How Much Should a Person Consume: Environmentalism in India and the United States

Ramachandra Guha

Berkeley:University of California Press, 2006. 276 pp. ISBN: 0-520-24803-1, $55

Inescapable Ecologies: A History of Environment, Disease, and Knowledge

Linda Nash

Berkeley:University of California Press, 2006. 346 pp. ISBN: 0-520-24891-0, $60

Nonparametric Functional Data Analysis: Theory and Practice

Frédéric Ferraty, Philippe Vieu

New York:Springer, 2006. 268 pp. ISBN: 0-387-30369-3, $79.95

Public Health: Innovation and Intellectual Property Rights

World Health Organization

Geneva:WHO Press, 2006. 216 pp. ISBN: 92-4-156323-0, $27

Statistical Analysis of Environmental Space–Time Processes

Nhu D. Le, James V. Zidek

New York:Springer, 2006. 341 pp. ISBN: 0-387-26209-1, $79.95

System Modeling in Cellular Biology

Zoltan Szallasi, Jörg Stelling, Vipul Periwal

Cambridge, MA:MIT Press, 2006. 480 pp. ISBN 0-262-19548-8, $50

The Atlas of Climate Change: Mapping the World’s Greatest Challenge

Kirstin Dow, Thomas E. Downing

Berkeley:University of California Press, 2006. 112 pp. ISBN: 0-520-25023-0, $19.95

Verifying Treaty Compliance: Limiting Weapons of Mass Destruction and Monitoring Kyoto Protocol Provisions

R. Avenhaus, N. Kyriakopoulos, M. Richard, G. Stein, eds.

New York:Springer, 2006. 629 pp. ISBN: 3-540-33853-5, $179

